# Supplementary material for: R-spondins can potentiate WNT signaling without LGRs
Source: eLife. 2018 Feb 6;7:e33126. doi: 10.7554/eLife.33126 (PMC5800842; doi:10.7554/eLife.33126)
Supplement: Supplementary file 2. — The name and length of each construct is indicated. Lowercase sequences overlap the sequence upstream of the unique AgeI site and downstream of the unique KpnI site in the pHLsec-HA-Tev-Fc-Avi-1D4 vector. Uppercase sequences encode human RSPO1 and RSPO3 WT, mutant and chimeric constructs. For point mutants, mutated codons are underlined. [file elife-33126-supp2.docx]

RSPO1 (WT): 791bp

cctacgacgtgcccgactacgccaccggtaacctgAGCCGGGGGATCAAGGGGAAAAGGCAGAGGCGGATCAGTGCCGAGGGGAGCCAGGCCTGTGCCAAAGGCTGTGAGCTCTGCTCTGAAGTCAACGGCTGCCTCAAGTGCTCACCCAAGCTGTTCATCCTGCTGGAGAGGAACGACATCCGCCAGGTGGGCGTCTGCTTGCCGTCCTGCCCACCTGGATACTTCGACGCCCGCAACCCCGACATGAACAAGTGCATCAAATGCAAGATCGAGCACTGTGAGGCCTGCTTCAGCCATAACTTCTGCACCAAGTGTAAGGAGGGCTTGTACCTGCACAAGGGCCGCTGCTATCCAGCTTGTCCCGAGGGCTCCTCAGCTGCCAATGGCACCATGGAGTGCAGTAGTCCTGCGCAATGTGAAATGAGCGAGTGGTCTCCGTGGGGGCCCTGCTCCAAGAAGCAGCAGCTCTGTGGTTTCCGGAGGGGCTCCGAGGAGCGGACACGCAGGGTGCTACATGCCCCTGTGGGGGACCATGCTGCCTGCTCTGACACCAAGGAGACCCGGAGGTGCACAGTGAGGAGAGTGCCGTGTCCTGAGGGGCAGAAGAGGAGGAAGGGAGGCCAGGGCCGGCGGGAGAATGCCAACAGGAACCTGGCCAGGAAGGAGAGCAAGGAGGCGGGTGCTGGCTCTCGAAGACGCAAGGGGCAGCAACAGCAGCAGCAGCAAGGGACAGTGGGGCCACTCACATCTGCAGGGCCTGCCttggtaccaaccaccgagaacctgtac

RSPO1 (R66A/Q71A): 791bp

cctacgacgtgcccgactacgccaccggtaacctgAGCCGGGGGATCAAGGGGAAAAGGCAGAGGCGGATCAGTGCCGAGGGGAGCCAGGCCTGTGCCAAAGGCTGTGAGCTCTGCTCTGAAGTCAACGGCTGCCTCAAGTGCTCACCCAAGCTGTTCATCCTGCTGGAGGCCAACGACATCCGCGCCGTGGGCGTCTGCTTGCCGTCCTGCCCACCTGGATACTTCGACGCCCGCAACCCCGACATGAACAAGTGCATCAAATGCAAGATCGAGCACTGTGAGGCCTGCTTCAGCCATAACTTCTGCACCAAGTGTAAGGAGGGCTTGTACCTGCACAAGGGCCGCTGCTATCCAGCTTGTCCCGAGGGCTCCTCAGCTGCCAATGGCACCATGGAGTGCAGTAGTCCTGCGCAATGTGAAATGAGCGAGTGGTCTCCGTGGGGGCCCTGCTCCAAGAAGCAGCAGCTCTGTGGTTTCCGGAGGGGCTCCGAGGAGCGGACACGCAGGGTGCTACATGCCCCTGTGGGGGACCATGCTGCCTGCTCTGACACCAAGGAGACCCGGAGGTGCACAGTGAGGAGAGTGCCGTGTCCTGAGGGGCAGAAGAGGAGGAAGGGAGGCCAGGGCCGGCGGGAGAATGCCAACAGGAACCTGGCCAGGAAGGAGAGCAAGGAGGCGGGTGCTGGCTCTCGAAGACGCAAGGGGCAGCAACAGCAGCAGCAGCAAGGGACAGTGGGGCCACTCACATCTGCAGGGCCTGCCttggtaccaaccaccgagaacctgtac

RSPO1 (F106E/F110E): 791bp

cctacgacgtgcccgactacgccaccggtaacctgAGCCGGGGGATCAAGGGGAAAAGGCAGAGGCGGATCAGTGCCGAGGGGAGCCAGGCCTGTGCCAAAGGCTGTGAGCTCTGCTCTGAAGTCAACGGCTGCCTCAAGTGCTCACCCAAGCTGTTCATCCTGCTGGAGAGGAACGACATCCGCCAGGTGGGCGTCTGCTTGCCGTCCTGCCCACCTGGATACTTCGACGCCCGCAACCCCGACATGAACAAGTGCATCAAATGCAAGATCGAGCACTGTGAGGCCTGCGAGAGCCATAACGAGTGCACCAAGTGTAAGGAGGGCTTGTACCTGCACAAGGGCCGCTGCTATCCAGCTTGTCCCGAGGGCTCCTCAGCTGCCAATGGCACCATGGAGTGCAGTAGTCCTGCGCAATGTGAAATGAGCGAGTGGTCTCCGTGGGGGCCCTGCTCCAAGAAGCAGCAGCTCTGTGGTTTCCGGAGGGGCTCCGAGGAGCGGACACGCAGGGTGCTACATGCCCCTGTGGGGGACCATGCTGCCTGCTCTGACACCAAGGAGACCCGGAGGTGCACAGTGAGGAGAGTGCCGTGTCCTGAGGGGCAGAAGAGGAGGAAGGGAGGCCAGGGCCGGCGGGAGAATGCCAACAGGAACCTGGCCAGGAAGGAGAGCAAGGAGGCGGGTGCTGGCTCTCGAAGACGCAAGGGGCAGCAACAGCAGCAGCAGCAAGGGACAGTGGGGCCACTCACATCTGCAGGGCCTGCCttggtaccaaccaccgagaacctgtac

RSPO1 (RSPO3 FU1): 791bp

cctacgacgtgcccgactacgccaccggtaacctgCAAAACGCCTCCCGGGGAAGGCGCCAGCGAAGAATGCATCCTAACGTTAGTCAAGGCTGCCAAGGAGGCTGTGCAACATGCTCAGATTACAATGGATGTTTGTCATGTAAGCCCAGACTATTTTTTGCTCTGGAAAGAATTGGCATGAAGCAGATTGGAGTATGTCTCTCTTCATGTCCAAGTGGATATTATGGAACTCGATATCCCGACATGAACAAGTGCATCAAATGCAAGATCGAGCACTGTGAGGCCTGCTTCAGCCATAACTTCTGCACCAAGTGTAAGGAGGGCTTGTACCTGCACAAGGGCCGCTGCTATCCAGCTTGTCCCGAGGGCTCCTCAGCTGCCAATGGCACCATGGAGTGCAGTAGTCCTGCGCAATGTGAAATGAGCGAGTGGTCTCCGTGGGGGCCCTGCTCCAAGAAGCAGCAGCTCTGTGGTTTCCGGAGGGGCTCCGAGGAGCGGACACGCAGGGTGCTACATGCCCCTGTGGGGGACCATGCTGCCTGCTCTGACACCAAGGAGACCCGGAGGTGCACAGTGAGGAGAGTGCCGTGTCCTGAGGGGCAGAAGAGGAGGAAGGGAGGCCAGGGCCGGCGGGAGAATGCCAACAGGAACCTGGCCAGGAAGGAGAGCAAGGAGGCGGGTGCTGGCTCTCGAAGACGCAAGGGGCAGCAACAGCAGCAGCAGCAAGGGACAGTGGGGCCACTCACATCTGCAGGGCCTGCCttggtaccaaccaccgagaacctgtac

RSPO1 (RSPO3 TSP): 791bp

cctacgacgtgcccgactacgccaccggtaacctgAGCCGGGGGATCAAGGGGAAAAGGCAGAGGCGGATCAGTGCCGAGGGGAGCCAGGCCTGTGCCAAAGGCTGTGAGCTCTGCTCTGAAGTCAACGGCTGCCTCAAGTGCTCACCCAAGCTGTTCATCCTGCTGGAGAGGAACGACATCCGCCAGGTGGGCGTCTGCTTGCCGTCCTGCCCACCTGGATACTTCGACGCCCGCAACCCCGACATGAACAAGTGCATCAAATGCAAGATCGAGCACTGTGAGGCCTGCTTCAGCCATAACTTCTGCACCAAGTGTAAGGAGGGCTTGTACCTGCACAAGGGCCGCTGCTATCCAGCTTGTCCCGAGGGCTCCTCAGCTGCCAATGGCACCATGGAGTGCAGTAGTATTGTGCACTGTGAGGTCAGTGAATGGAATCCTTGGAGTCCATGCACGAAGAAGGGAAAAACATGTGGCTTCAAAAGAGGGACTGAAACACGGGTCCGAGAAATAATACAGCATCCTTCAGCAAAGGGTAACCTGTGTCCCCCAACAAATGAGACAAGAAAGTGTACAGTGCAAAGGAAGAAGTGTCAGAAGGGGCAGAAGAGGAGGAAGGGAGGCCAGGGCCGGCGGGAGAATGCCAACAGGAACCTGGCCAGGAAGGAGAGCAAGGAGGCGGGTGCTGGCTCTCGAAGACGCAAGGGGCAGCAACAGCAGCAGCAGCAAGGGACAGTGGGGCCACTCACATCTGCAGGGCCTGCCttggtaccaaccaccgagaacctgtac

RSPO1 (RSPO3 BR): 818bp

cctacgacgtgcccgactacgccaccggtaacctgAGCCGGGGGATCAAGGGGAAAAGGCAGAGGCGGATCAGTGCCGAGGGGAGCCAGGCCTGTGCCAAAGGCTGTGAGCTCTGCTCTGAAGTCAACGGCTGCCTCAAGTGCTCACCCAAGCTGTTCATCCTGCTGGAGAGGAACGACATCCGCCAGGTGGGCGTCTGCTTGCCGTCCTGCCCACCTGGATACTTCGACGCCCGCAACCCCGACATGAACAAGTGCATCAAATGCAAGATCGAGCACTGTGAGGCCTGCTTCAGCCATAACTTCTGCACCAAGTGTAAGGAGGGCTTGTACCTGCACAAGGGCCGCTGCTATCCAGCTTGTCCCGAGGGCTCCTCAGCTGCCAATGGCACCATGGAGTGCAGTAGTCCTGCGCAATGTGAAATGAGCGAGTGGTCTCCGTGGGGGCCCTGCTCCAAGAAGCAGCAGCTCTGTGGTTTCCGGAGGGGCTCCGAGGAGCGGACACGCAGGGTGCTACATGCCCCTGTGGGGGACCATGCTGCCTGCTCTGACACCAAGGAGACCCGGAGGTGCACAGTGAGGAGAGTGCCGTGTCCTGAGGGGGAACGAGGAAAAAAAGGAAGGGAGAGGAAAAGAAAAAAACCTAATAAAGGAGAAAGTAAAGAAGCAATACCTGACAGCAAAAGTCTGGAATCCAGCAAAGAAATCCCAGAGCAACGAGAAAACAAACAGCAGCAGAAGAAGCGAAAAGTCCAAGATAAACAGAAATCGGTATCAGTCAGCACTGTACACttggtaccaaccaccgagaacctgtac

RSPO1 (RSPO3 TSP/BR): 818bp

cctacgacgtgcccgactacgccaccggtaacctgAGCCGGGGGATCAAGGGGAAAAGGCAGAGGCGGATCAGTGCCGAGGGGAGCCAGGCCTGTGCCAAAGGCTGTGAGCTCTGCTCTGAAGTCAACGGCTGCCTCAAGTGCTCACCCAAGCTGTTCATCCTGCTGGAGAGGAACGACATCCGCCAGGTGGGCGTCTGCTTGCCGTCCTGCCCACCTGGATACTTCGACGCCCGCAACCCCGACATGAACAAGTGCATCAAATGCAAGATCGAGCACTGTGAGGCCTGCTTCAGCCATAACTTCTGCACCAAGTGTAAGGAGGGCTTGTACCTGCACAAGGGCCGCTGCTATCCAGCTTGTCCCGAGGGCTCCTCAGCTGCCAATGGCACCATGGAGTGCAGTAGTATTGTGCACTGTGAGGTCAGTGAATGGAATCCTTGGAGTCCATGCACGAAGAAGGGAAAAACATGTGGCTTCAAAAGAGGGACTGAAACACGGGTCCGAGAAATAATACAGCATCCTTCAGCAAAGGGTAACCTGTGTCCCCCAACAAATGAGACAAGAAAGTGTACAGTGCAAAGGAAGAAGTGTCAGAAGGGAGAACGAGGAAAAAAAGGAAGGGAGAGGAAAAGAAAAAAACCTAATAAAGGAGAAAGTAAAGAAGCAATACCTGACAGCAAAAGTCTGGAATCCAGCAAAGAAATCCCAGAGCAACGAGAAAACAAACAGCAGCAGAAGAAGCGAAAAGTCCAAGATAAACAGAAATCGGTATCAGTCAGCACTGTACACttggtaccaaccaccgagaacctgtac

RSPO3 (WT): 815bp

cctacgacgtgcccgactacgccaccggtaacctgCAAAACGCCTCCCGGGGAAGGCGCCAGCGAAGAATGCATCCTAACGTTAGTCAAGGCTGCCAAGGAGGCTGTGCAACATGCTCAGATTACAATGGATGTTTGTCATGTAAGCCCAGACTATTTTTTGCTCTGGAAAGAATTGGCATGAAGCAGATTGGAGTATGTCTCTCTTCATGTCCAAGTGGATATTATGGAACTCGATATCCAGATATAAATAAGTGTACAAAATGCAAAGCTGACTGTGATACCTGTTTCAACAAAAATTTCTGCACAAAATGTAAAAGTGGATTTTACTTACACCTTGGAAAGTGCCTTGACAATTGCCCAGAAGGGTTGGAAGCCAACAACCATACTATGGAGTGTGTCAGTATTGTGCACTGTGAGGTCAGTGAATGGAATCCTTGGAGTCCATGCACGAAGAAGGGAAAAACATGTGGCTTCAAAAGAGGGACTGAAACACGGGTCCGAGAAATAATACAGCATCCTTCAGCAAAGGGTAACCTGTGTCCCCCAACAAATGAGACAAGAAAGTGTACAGTGCAAAGGAAGAAGTGTCAGAAGGGAGAACGAGGAAAAAAAGGAAGGGAGAGGAAAAGAAAAAAACCTAATAAAGGAGAAAGTAAAGAAGCAATACCTGACAGCAAAAGTCTGGAATCCAGCAAAGAAATCCCAGAGCAACGAGAAAACAAACAGCAGCAGAAGAAGCGAAAAGTCCAAGATAAACAGAAATCGGTATCAGTCAGCACTGTACACttggtaccaaccaccgagaacctgtac

RSPO3 ΔFU1/FU2: 449bp

cctacgacgtgcccgactacgccaccggtaacctgAGTATTGTGCACTGTGAGGTCAGTGAATGGAATCCTTGGAGTCCATGCACGAAGAAGGGAAAAACATGTGGCTTCAAAAGAGGGACTGAAACACGGGTCCGAGAAATAATACAGCATCCTTCAGCAAAGGGTAACCTGTGTCCCCCAACAAATGAGACAAGAAAGTGTACAGTGCAAAGGAAGAAGTGTCAGAAGGGAGAACGAGGAAAAAAAGGAAGGGAGAGGAAAAGAAAAAAACCTAATAAAGGAGAAAGTAAAGAAGCAATACCTGACAGCAAAAGTCTGGAATCCAGCAAAGAAATCCCAGAGCAACGAGAAAACAAACAGCAGCAGAAGAAGCGAAAAGTCCAAGATAAACAGAAATCGGTATCAGTCAGCACTGTACACttggtaccaaccaccgagaacctgtac

RSPO3 ΔTSP: 632bp

cctacgacgtgcccgactacgccaccggtaacctgCAAAACGCCTCCCGGGGAAGGCGCCAGCGAAGAATGCATCCTAACGTTAGTCAAGGCTGCCAAGGAGGCTGTGCAACATGCTCAGATTACAATGGATGTTTGTCATGTAAGCCCAGACTATTTTTTGCTCTGGAAAGAATTGGCATGAAGCAGATTGGAGTATGTCTCTCTTCATGTCCAAGTGGATATTATGGAACTCGATATCCAGATATAAATAAGTGTACAAAATGCAAAGCTGACTGTGATACCTGTTTCAACAAAAATTTCTGCACAAAATGTAAAAGTGGATTTTACTTACACCTTGGAAAGTGCCTTGACAATTGCCCAGAAGGGTTGGAAGCCAACAACCATACTATGGAGTGTGTCAGTATTGTGAAGGGAGAACGAGGAAAAAAAGGAAGGGAGAGGAAAAGAAAAAAACCTAATAAAGGAGAAAGTAAAGAAGCAATACCTGACAGCAAAAGTCTGGAATCCAGCAAAGAAATCCCAGAGCAACGAGAAAACAAACAGCAGCAGAAGAAGCGAAAAGTCCAAGATAAACAGAAATCGGTATCAGTCAGCACTGTACACttggtaccaaccaccgagaacctgtac

RSPO3 ΔBR: 620bp

cctacgacgtgcccgactacgccaccggtaacctgCAAAACGCCTCCCGGGGAAGGCGCCAGCGAAGAATGCATCCTAACGTTAGTCAAGGCTGCCAAGGAGGCTGTGCAACATGCTCAGATTACAATGGATGTTTGTCATGTAAGCCCAGACTATTTTTTGCTCTGGAAAGAATTGGCATGAAGCAGATTGGAGTATGTCTCTCTTCATGTCCAAGTGGATATTATGGAACTCGATATCCAGATATAAATAAGTGTACAAAATGCAAAGCTGACTGTGATACCTGTTTCAACAAAAATTTCTGCACAAAATGTAAAAGTGGATTTTACTTACACCTTGGAAAGTGCCTTGACAATTGCCCAGAAGGGTTGGAAGCCAACAACCATACTATGGAGTGTGTCAGTATTGTGCACTGTGAGGTCAGTGAATGGAATCCTTGGAGTCCATGCACGAAGAAGGGAAAAACATGTGGCTTCAAAAGAGGGACTGAAACACGGGTCCGAGAAATAATACAGCATCCTTCAGCAAAGGGTAACCTGTGTCCCCCAACAAATGAGACAAGAAAGTGTACAGTGCAAAGGAAGAAGTGTCAGttggtaccaaccaccgagaacctgtac

RSPO3 ΔTSP/BR: 437bp

cctacgacgtgcccgactacgccaccggtaacctgCAAAACGCCTCCCGGGGAAGGCGCCAGCGAAGAATGCATCCTAACGTTAGTCAAGGCTGCCAAGGAGGCTGTGCAACATGCTCAGATTACAATGGATGTTTGTCATGTAAGCCCAGACTATTTTTTGCTCTGGAAAGAATTGGCATGAAGCAGATTGGAGTATGTCTCTCTTCATGTCCAAGTGGATATTATGGAACTCGATATCCAGATATAAATAAGTGTACAAAATGCAAAGCTGACTGTGATACCTGTTTCAACAAAAATTTCTGCACAAAATGTAAAAGTGGATTTTACTTACACCTTGGAAAGTGCCTTGACAATTGCCCAGAAGGGTTGGAAGCCAACAACCATACTATGGAGTGTGTCAGTATTGTGttggtaccaaccaccgagaacctgtac

RSPO3 (R67A/Q72A): 815bp

cctacgacgtgcccgactacgccaccggtaacctgCAAAACGCCTCCCGGGGAAGGCGCCAGCGAAGAATGCATCCTAACGTTAGTCAAGGCTGCCAAGGAGGCTGTGCAACATGCTCAGATTACAATGGATGTTTGTCATGTAAGCCCAGACTATTTTTTGCTCTGGAAGCCATTGGCATGAAGGCCATTGGAGTATGTCTCTCTTCATGTCCAAGTGGATATTATGGAACTCGATATCCAGATATAAATAAGTGTACAAAATGCAAAGCTGACTGTGATACCTGTTTCAACAAAAATTTCTGCACAAAATGTAAAAGTGGATTTTACTTACACCTTGGAAAGTGCCTTGACAATTGCCCAGAAGGGTTGGAAGCCAACAACCATACTATGGAGTGTGTCAGTATTGTGCACTGTGAGGTCAGTGAATGGAATCCTTGGAGTCCATGCACGAAGAAGGGAAAAACATGTGGCTTCAAAAGAGGGACTGAAACACGGGTCCGAGAAATAATACAGCATCCTTCAGCAAAGGGTAACCTGTGTCCCCCAACAAATGAGACAAGAAAGTGTACAGTGCAAAGGAAGAAGTGTCAGAAGGGAGAACGAGGAAAAAAAGGAAGGGAGAGGAAAAGAAAAAAACCTAATAAAGGAGAAAGTAAAGAAGCAATACCTGACAGCAAAAGTCTGGAATCCAGCAAAGAAATCCCAGAGCAACGAGAAAACAAACAGCAGCAGAAGAAGCGAAAAGTCCAAGATAAACAGAAATCGGTATCAGTCAGCACTGTACACttggtaccaaccaccgagaacctgtac

RSPO3 (F106E/F110E): 815bp

cctacgacgtgcccgactacgccaccggtaacctgCAAAACGCCTCCCGGGGAAGGCGCCAGCGAAGAATGCATCCTAACGTTAGTCAAGGCTGCCAAGGAGGCTGTGCAACATGCTCAGATTACAATGGATGTTTGTCATGTAAGCCCAGACTATTTTTTGCTCTGGAAAGAATTGGCATGAAGCAGATTGGAGTATGTCTCTCTTCATGTCCAAGTGGATATTATGGAACTCGATATCCAGATATAAATAAGTGTACAAAATGCAAAGCTGACTGTGATACCTGTGAGAACAAAAATGAGTGCACAAAATGTAAAAGTGGATTTTACTTACACCTTGGAAAGTGCCTTGACAATTGCCCAGAAGGGTTGGAAGCCAACAACCATACTATGGAGTGTGTCAGTATTGTGCACTGTGAGGTCAGTGAATGGAATCCTTGGAGTCCATGCACGAAGAAGGGAAAAACATGTGGCTTCAAAAGAGGGACTGAAACACGGGTCCGAGAAATAATACAGCATCCTTCAGCAAAGGGTAACCTGTGTCCCCCAACAAATGAGACAAGAAAGTGTACAGTGCAAAGGAAGAAGTGTCAGAAGGGAGAACGAGGAAAAAAAGGAAGGGAGAGGAAAAGAAAAAAACCTAATAAAGGAGAAAGTAAAGAAGCAATACCTGACAGCAAAAGTCTGGAATCCAGCAAAGAAATCCCAGAGCAACGAGAAAACAAACAGCAGCAGAAGAAGCGAAAAGTCCAAGATAAACAGAAATCGGTATCAGTCAGCACTGTACACttggtaccaaccaccgagaacctgtac

RSPO3 (RSPO1 FU1): 815bp

cctacgacgtgcccgactacgccaccggtaacctgAGCCGGGGGATCAAGGGGAAAAGGCAGAGGCGGATCAGTGCCGAGGGGAGCCAGGCCTGTGCCAAAGGCTGTGAGCTCTGCTCTGAAGTCAACGGCTGCCTCAAGTGCTCACCCAAGCTGTTCATCCTGCTGGAGAGGAACGACATCCGCCAGGTGGGCGTCTGCTTGCCGTCCTGCCCACCTGGATACTTCGACGCCCGCAACCCAGATATAAATAAGTGTACAAAATGCAAAGCTGACTGTGATACCTGTTTCAACAAAAATTTCTGCACAAAATGTAAAAGTGGATTTTACTTACACCTTGGAAAGTGCCTTGACAATTGCCCAGAAGGGTTGGAAGCCAACAACCATACTATGGAGTGTGTCAGTATTGTGCACTGTGAGGTCAGTGAATGGAATCCTTGGAGTCCATGCACGAAGAAGGGAAAAACATGTGGCTTCAAAAGAGGGACTGAAACACGGGTCCGAGAAATAATACAGCATCCTTCAGCAAAGGGTAACCTGTGTCCCCCAACAAATGAGACAAGAAAGTGTACAGTGCAAAGGAAGAAGTGTCAGAAGGGAGAACGAGGAAAAAAAGGAAGGGAGAGGAAAAGAAAAAAACCTAATAAAGGAGAAAGTAAAGAAGCAATACCTGACAGCAAAAGTCTGGAATCCAGCAAAGAAATCCCAGAGCAACGAGAAAACAAACAGCAGCAGAAGAAGCGAAAAGTCCAAGATAAACAGAAATCGGTATCAGTCAGCACTGTACACttggtaccaaccaccgagaacctgtac

RSPO3 (RSPO1 TSP/BR): 788bp

cctacgacgtgcccgactacgccaccggtaacctgCAAAACGCCTCCCGGGGAAGGCGCCAGCGAAGAATGCATCCTAACGTTAGTCAAGGCTGCCAAGGAGGCTGTGCAACATGCTCAGATTACAATGGATGTTTGTCATGTAAGCCCAGACTATTTTTTGCTCTGGAAAGAATTGGCATGAAGCAGATTGGAGTATGTCTCTCTTCATGTCCAAGTGGATATTATGGAACTCGATATCCAGATATAAATAAGTGTACAAAATGCAAAGCTGACTGTGATACCTGTTTCAACAAAAATTTCTGCACAAAATGTAAAAGTGGATTTTACTTACACCTTGGAAAGTGCCTTGACAATTGCCCAGAAGGGTTGGAAGCCAACAACCATACTATGGAGTGTGTCAGTCCTGCGCAATGTGAAATGAGCGAGTGGTCTCCGTGGGGGCCCTGCTCCAAGAAGCAGCAGCTCTGTGGTTTCCGGAGGGGCTCCGAGGAGCGGACACGCAGGGTGCTACATGCCCCTGTGGGGGACCATGCTGCCTGCTCTGACACCAAGGAGACCCGGAGGTGCACAGTGAGGAGAGTGCCGTGTCCTGAGGGGCAGAAGAGGAGGAAGGGAGGCCAGGGCCGGCGGGAGAATGCCAACAGGAACCTGGCCAGGAAGGAGAGCAAGGAGGCGGGTGCTGGCTCTCGAAGACGCAAGGGGCAGCAACAGCAGCAGCAGCAAGGGACAGTGGGGCCACTCACATCTGCAGGGCCTGCCttggtaccaaccaccgagaacctgtac
